# Science evolves but outdated testing and static risk management in the US delay protection to human health

**DOI:** 10.3389/ftox.2024.1444024

**Published:** 2024-08-13

**Authors:** Maricel V. Maffini, Laura N. Vandenberg

**Affiliations:** ^1^ Independent Consultant, Frederick, MD, United States; ^2^ School of Public Health and Health Sciences, University of Massachusetts – Amherst, Amherst, MA, United States

**Keywords:** US Food and Drug Administration, US Environmental Protection Agency, hazard assessment, per-and polyfluorinated alkyl substances, regulation

## Introduction

There is increasing concern amongst public health professionals, environmental health scientists, and medical organizations about exposures to synthetic chemicals (Deborah Bennett et al., 2016; Bergman et al., 2013a; Di Renzo et al., 2015; Gore et al., 2015; Persson et al., 2022; Trasande et al., 2018) via polluted air and water, consumer products such as cosmetics, fabrics and upholstery, and food and packaging. These organizations’ concerns are based on the overwhelming evidence showing associations between chemical exposures and adverse health outcomes in human populations.

Some of the strongest evidence has come from persistent organic pollutants that bioaccumulate in the bodies of animals and people and bio magnify in the food chain. For example, per- and polyfluorinated alkyl substances, aka PFAS, have received significant attention following the C8 Health Project which was created after communities in Parkersburg, West Virginia (US) were affected by contamination of drinking water from the manufacturer of perfluorooctanoic acid (PFOA). People in these communities sued the manufacturer and, through a settlement agreement, medical monitoring of the population was developed (Frisbee Stephanie et al., 2009). The C8 Health Project and subsequent studies have revealed associations between PFOA exposures and human health effects including cardiometabolic issues, thyroid disorders, kidney and testicular cancer, and ulcerative colitis (Steenland et al., 2020).

Evidence accumulated over several decades also showed adverse effects associated with exposures to non-persistent chemicals including chemicals used in plastics, personal care products, solvents, detergents, food dyes, and pesticide ingredients (Zoeller et al., 2014; Skakkebaek et al., 2016; La Merrill et al., 2020; Vom Saal and Vandenberg, 2021; Matouskova and Vandenberg, 2022; Stacy et al., 2024). These chemicals are widely used in consumer products, and are so ubiquitous that they are considered pseudo-persistent, i.e., producing continuous exposures from external sources (Barceló and Petrovic, 2007). There are now thousands of studies showing associations between these chemicals and adverse health effects in humans including neurological disorders and learning disabilities, metabolic outcomes, infertility, thyroid dysfunction, and cancers (Bergman et al., 2013b; de Graaf et al., 2022; Gore et al., 2015; Hamed et al., 2023; Heindel et al., 2017; Miller et al., 2022; Muncke et al., 2020; Onyije et al., 2024; Patisaul, 2021; Rancière et al., 2015; Ribeiro et al., 2020; Wu et al., 2023; Zoeller et al., 2012).

The growing evidence linking chemical exposures to chronic diseases has led experts to deem the testing approaches recommended by regulatory agencies for risk assessment (EPA, 2023; FDA, 2000) insufficient to protect human health (Bergman et al., 2013a; Bennett et al., 2016; Anderko, 2017; Prins et al., 2019; Vandenberg, 2019; Vom Saal, 2019; Vandenberg, 2021b; Kassotis et al., 2022). Many endpoints such as developmental neurotoxicity, immunotoxicity, endocrine disruption and non-genotoxic carcinogenicity lack appropriate assays to protect human health. Testing that relies on more sensitive and health-relevant endpoints would reduce or eliminate exposures to hazardous chemicals before they enter the marketplace (Zimmermann et al., 2022).

In this commentary, we describe what better testing approaches for use in risk assessment would look like and how improvements in these tests would positively impact human health. We also discuss how inadequate risk management approaches lead to insufficient protections of human health. Although our focus is mostly on the United States, our conclusions are generally applicable to other countries.

### What does better testing look like?

Although the exact number of chemicals remains unknown, scientists estimate there are 140,000–300,000 chemicals on the global market (Wang et al., 2020). In the US more than 42,000 chemicals listed on the US Environmental Protection Agency’s Toxic Substances Control Act (TSCA) inventory are currently in use in consumer products or in industrial processes. Furthermore, worldwide there are more than 12,000 chemicals authorized to use in the manufacturing of materials in contact with food (Groh et al., 2021) and the US Food and Drug Administration (FDA) has authorized more than 10,000 chemicals to be directly used in food or in food packaging and processing equipment (Neltner et al., 2011). The identity of many of these chemicals remains unknown because they are shielded as “confidential business information” or because they are registered in other countries without public disclosures (Vandenberg et al., 2023). One problem with the current approach to chemical testing in the US is that the data gaps for these chemicals are extensive. For instance, evaluations of ingredients added to food indicate that less than 25% have a feeding study that can be used to calculate safe levels of exposure and less than 10% have either reproductive or developmental toxicity data available (Neltner et al., 2013). Others have also reported on the lack of data for food ingredients (Faustman et al., 2021; Matouskova et al., 2023).

With the number of chemicals currently on the market, the problem is not only that we have failed to test a large number of them prior to their authorization, but that often the hazards of these chemicals are revealed years after they enter commerce, and in the US, there are very few options to restrict the use of chemicals once they have been allowed in products. With the exception of pesticide ingredients which are routinely reevaluated by the US EPA, chemicals used in food packaging, consumer products, and industrial processes do not undergo post-market re-evaluation, so even when studies reveal harmful effects of exposures to these chemicals, the options to restrict their uses are limited. This means that pre-market testing is critical to protect the health of humans and the environment.

To address these problems collectively, we need reliable assays that can be used for risk assessment and regulatory purposes (Cediel-Ulloa et al., 2022; Legler et al., 2020; Schug et al., 2013a; Schug et al., 2013b; Street et al., 2021; Vandenberg et al., 2019; Vandenberg, 2021a; b). It is not sufficient to develop *in vitro* screening tests like those identified as new approach methodologies (NAMs), it is also necessary to demonstrate that those NAMs are as good, if not better, than modern mammalian tests at identifying hazards (Kortenkamp et al., 2020; De Castelbajac et al., 2023; Tal et al., 2024). NAMs also need to go through validation processes to show that they are reproducible in other groups. Also, assays should accurately identify exposure levels where adverse effects do not occur. Lastly, regulators are expected to use data from assays, including NAMs, to protect the public’s health rather than protecting chemicals from further scrutiny. None of these has been done successfully to date.

Better testing would also use class-based approaches, like those that are required of the FDA but that have not been implemented (Maffini et al., 2023); with this approach, data from a few chemicals can be used to regulate others in the same class before they reach the market. As there is increasing evidence that chemicals in the food supply and in consumer products cause harm (Maffini and Vandenberg, 2017; Groh et al., 2019; Muncke, 2021), there needs to be evidence-based periodic post-market reevaluations and updated risk management decisions to remove the bad actors without introducing regrettable replacements (Woodruff et al., 2023).

Another issue is that many of the standard assays used to evaluate some hazards (often described in internationally-recognized test guidelines) focus on signs of acute toxicity rather than outcomes that are relevant to chronic diseases and conditions that are increasing in human populations (Lupu et al., 2020; Vandenberg, 2021a; b). For example, there are limited approaches to determine if chemicals affect the breast, even though there is increasing evidence that girls are experiencing premature breast development, increasing reports of shortened duration of breastfeeding in women that want to nurse, and increasing rates of premenopausal breast cancers (Kay et al., 2022). Although mammary glands are collected in some rodent toxicity tests, mammary gland development and function remains understudied in toxicity tests (Matouskova et al., 2022). To date, relatively few high-throughput *in vitro* approaches, including NAMs, have been developed that focus on chronic noncommunicable diseases, but this is critical considering these conditions are the most important challenges to the health of modern human populations (Groh and Muncke, 2017; Muncke et al., 2023).

Human studies finding associations between early life exposure to chemicals indicate that toxicity testing should focus on health-related outcomes rather than overt signs of toxicity. Examples of concerning early-life exposures from epidemiology studies are dichlorodiphenyltrichloroethane (DDT) and later life breast cancer risk (Cohn et al., 2015; Cohn et al., 2019), perchlorate and diminished IQ levels in children (Steinmaus et al., 2010; Brent, 2014; Taylor et al., 2014), and bisphenol A (BPA) and increased risk of asthma in children (Xie et al., 2016; Wu et al., 2021; Abellan et al., 2022). Good testing approaches should expand the endpoints to include human-health relevant outcomes. If this can be done within the context of NAMs, it will help to speed up the evaluation process, but many of these outcomes are complex and will be challenging to assess outside of whole animals.

Current hazard identification approaches continue to rely on outdated principles and expectations. For example, common testing approaches assume that chemicals are quickly eliminated from the body, something that many PFAS and other persistent organic pollutants have disproven, even considering species-specific differences in their half-lives (Olsen et al., 2007). In fact, this assumption continues to create problems in the testing (and risk management) of shorter-chain PFAS, which were assumed to be less bioaccumulative, and thus less hazardous, than the long-chain PFAS. Unfortunately, this was revealed to be untrue (Kabadi et al., 2018; Rice et al., 2020; Rice et al., 2023). Another long-held assumption is that chemical metabolites are less hazardous than the parent compounds. Phthalates, which have several metabolites that are more biologically active than the parent compounds, have disproven this assumption as well (Zhang et al., 2021).

Current approaches also rely on the assumption that testing chemicals one at a time is appropriate to understand how chemicals act under real-world conditions. Numerous mixture studies, including ones that demonstrated cumulative effects, have disproven this assumption (Christiansen et al., 2020; Martin et al., 2021; Caporale et al., 2022). For example, studies combining chemicals at concentrations that were 80-fold lower than their individual lowest-observed-adverse-effect-levels can act together to induce malformations of the male reproductive tract (Conley et al., 2018). Human mixture studies focused on real-world mixtures from human biomonitoring have revealed that some chemicals drive disease risk more than others (Escher et al., 2022; Luijten et al., 2023). Importantly, these chemicals are used in different kinds of products (e.g., consumer products, cosmetics, industrial products, food packaging) and thus are regulated very differently.

Lastly, testing on adult animals (or in cultured cells) has been assumed to predict effects on developing animals. Numerous examples of environmental chemicals including many endocrine disrupting chemicals have shown this to be false (Balbus et al., 2013; Grandjean et al., 2015; Heindel and Vandenberg, 2015; Treviño and Katz, 2018). Rather, significant evidence suggests that early life exposures to chemicals can have profound, unique, and lasting effects on individuals (Bourguignon et al., 2013; Di Pietro et al., 2023) and future generations (Sargis et al., 2019).

To address these challenges, long-held assumptions that have driven risk assessment and regulations for more than half a century should be complemented—if not completely replaced—with modern scientific principles of toxicology including mixture toxicology, endocrinology, physiology, and immunology (Vandenberg et al., 2013). Testing needs to be nimbler to account for the growth in knowledge of these fields over the last three decades and the new knowledge that is yet to come as well as the complexity of chemical exposures and new chemistries (Arthur et al., 2015a; Arthur et al., 2015b).

### Improved testing leads to better risk management

The results of testing for hazard identification, like those described above, are used for risk assessment (Beronius et al., 2009). The National Academies (National Academies of Science, 1983) define risk assessment as “the use of the factual base to define the health effects of exposure of individuals or populations to hazardous materials and situations.” Risk assessment involves the combination of hazard, exposure, and dose response data to quantify the probability of an adverse effect at a specific level of exposure. After a risk assessment is performed, the next step is to decide whether the risk to health is substantial enough that it must be managed. Risk management is defined as “the process of weighing policy alternatives and selecting the most appropriate regulatory action, integrating the results of risk assessment with engineering data and with social, economic, and political concerns to reach a decision.”

Regulation is a common tool to manage risk. Regulations should allow chemicals to be used safely and appropriately through use limitations and restrictions. Regulations are bound by jurisdictional laws which means that the same chemical is regulated differently if it is used in toys than in food, even though the hazards remain the same. The legislative bodies writing these laws (e.g., at federal or state level in the US) are often slow to act, putting the public’s health at risk. But even when laws regulating chemicals are available, regulatory agencies then interpret and implement those laws through rules and regulations that can take broad or narrow approaches to protect the public. For example, chemical restrictions themselves can vary in severity, with bans as the most protective measure. A ban can include a total restriction in use of individual chemicals (methylene chloride in paint strippers (EPA, 2024); an entire class of chemicals (e.g., PFAS in food packaging (FDA, 2016; State, 2018); or groups of chemicals with similar adverse effects (e.g., anti-androgenic phthalates in children’s toys (Consumer Product Safety Commission, 2017). The least protective risk management is based on good manufacturing practice, which means that a chemical is permitted to be used at concentrations needed for the final article to perform properly, but no more (FDA, 1977). In general, the amounts that are actually used in the product are only known to the manufacturer. Other restrictions include specific migration limits (e.g., the amount of a chemical or mixture of chemicals that is allowed to be released from the product into food); restrictions that are focused on specific vulnerable populations (e.g., the restriction of a chemical in infant formula (FDA 2024a); consumption limits per day or per week that will not increase health risks (EFSA Panel on Food Contact Materials et al., 2023); and limits to the amount of a chemical added to the final article expressed by weight (FDA, 2005).

As stated above, risk assessment informs risk management (National Research Council, Division on Earth, Life Studies, Board on Environmental Studies, and Committee on Improving Risk Analysis Approaches Used by the US EPA, 2009) which may also consider economic cost to the regulated industry, availability of safer substitutes, societal values, political will and the precautionary principle. In an ideal world, risk assessment and risk management should be performed by different groups of experts (Maffini and Birnbaum, 2024) to ensure that the risk assessment is solely based on scientific evidence and is not influenced by the “costs” of taking action. This separation of risk assessment and risk management is certainly feasible, since this is the approach taken in the EU; for example, risk assessment for chemicals used in food and food packaging is conducted by the European Food Safety Authority whereas risk management decisions are the task of the European Commission. The subjective nature of risk management lends itself to criticism especially when it dismisses or disregards the risk assessment conclusions, and challenging a management decision usually causes delays in public health protection. Examples of delays include pesticides such as chlorpyrifos (Trasande, 2017) and glyphosate (Vandenberg et al., 2017), as well as chemicals in consumer products such as phthalates in food contact materials (Edwards, 2023) and building materials (asbestos) (Järvholm and Burdorf, 2024; Phillips, 2024). Unfortunately, risk management is often dependent on the strength of political will.

### A role for civil society

When testing is insufficient to identify the most concerning hazards associated with a chemical, and risk management strategies fail to protect vulnerable populations from concerning exposures, the public is left to act. Commonly used tools include educational campaigns to move consumers away from products that have chemicals of concern (Defend Our Health, 2017). Another option is to submit petitions to the relevant agency arguing the there is strong evidence a chemical presents a high risk to health, the lack of a risk assessment or the use of the chemical not meeting the standard of safety (FDA 2024b). An example of this is a petition that require FDA to demonstrate the safety of long-chain PFAS in food packaging, which the agency responded to by removing approval of three types of long-chain PFAS (FDA, 2016). Members of civil society can also sue a regulatory agency for not implementing a protective law (Center for Food Safety, 2022) or product manufacturers when experts have assembled sufficient evidence, meeting a legal burden of proof, that a chemical causes serious harm. For example, repeated lawsuits against manufacturers of herbicides containing glyphosate have been successful because of strong evidence these products increase the risk of Non-Hodgkin’s Lymphoma (Zhang et al., 2019), even though the EPA maintains that glyphosate does not cause cancer (Benbrook, 2019).

These examples show that the public is a powerful force to push regulators and the regulated community to address problematic chemicals. The role of civil society is especially critical when risk management decisions lag for years or lack sufficient teeth to protect the public’s health. Similarly, citizens have been willing to take action when academic studies and epidemiology findings reveal harmful effects of chemicals, even if those outcomes are not evaluated in traditional test guidelines or accounted for in an agency’s risk assessment and risk management decisions.

## Conclusion

We must be able to live with risk; nothing in our world is absent of risk. The problems we describe here illustrate a common paradox in US regulatory agencies: they are mandated to make safety decisions based on science that is constantly evolving while the risk management is commonly static. In other words, periodic reassessment of decisions based on new scientific evidence is not common in the US, with the exception of pesticides.

We argue that better testing will result in better risk assessments, and with better risk assessment, there is an opportunity for better risk management. Better testing, and better use of testing data, can protect the public’s health. However, this is not a given. Risk management involves numerous subjective factors including political will, which is often lacking.
